# Biocompatibility and Antitumor Effects of Magnetically Targeted L‑Cysteine-Functionalized Superparamagnetic Iron Oxide Nanoparticles in an Ehrlich Tumor Model

**DOI:** 10.1021/acsomega.6c01138

**Published:** 2026-07-17

**Authors:** Emerson Barbosa da Silva, Tatiane Nassar Britos, Camila dos Santos Chagas, Giuliana Petri, Glaucia Raquel Luciano da Veiga, Beatriz da Costa Aguiar Alves Reis, Paula Silvia Haddad, Fabio Furlan Ferreira, Fernando Luiz Affonso Fonseca

**Affiliations:** † Department of Pathology, 125191 Centro Universitário FMABC (FMABC), Santo André, São Paulo 09060-650, Brazil; ‡ Experimental Biotery Laboratory of the FMABC, Centro Universitário FMABC (FMABC), Santo André 09060-650, Brazil; § Department of Chemistry, 28105 Federal University of São Paulo (UNIFESP), Diadema, São Paulo 04021-001, Brazil; ∥ Center for Natural and Human Science (CCNH), 74362 Federal University of ABC (UFABC), Santo André, São Paulo 09280-560, Brazil; ⊥ Department of Pharmaceutical Sciences, Federal University of São Paulo (UNIFESP), Diadema Campus, Diadema, São Paulo 04021-001, Brazil

## Abstract

Superparamagnetic iron oxide nanoparticles (SPIONs-Fe_3_O_4_) functionalized with l-cysteine (SPIONs-l-Cys) were evaluated as a biocompatible, magnetically responsive nanosystem in an Ehrlich solid tumor model. The formulation was obtained by chemical coprecipitation, and its complementary physicochemical characterization is provided in the Supporting Information and supported by our previous report on the same l-cysteine-functionalized magnetite system, including X-ray diffraction, FTIR, hydrodynamic size, polydispersity index, ζ-potential, surface thiol quantification by DTNB, magnetic measurements, and complementary in vitro data. The supplementary characterization supports the preservation of the magnetite crystalline structure after functionalization and confirms the presence of free thiol groups in an aqueous dispersion. *In vitro* cytotoxicity assays using human mononuclear cells and Ehrlich tumor cells indicated low toxicity under selected experimental conditions and biologically relevant redox-associated activities. *In vivo* evaluation demonstrated preserved hematological and biochemical parameters, the absence of overt systemic toxicity, and reduced tumor burden in groups treated with SPIONs-l-Cys under magnetic targeting conditions. These findings support the biological compatibility of SPIONs-l-Cys and indicate antitumor-associated effects under localized magnetic field conditions, while not establishing intratumoral accumulation, drug delivery, or a definitive mechanism of action.

## Graphical abstract

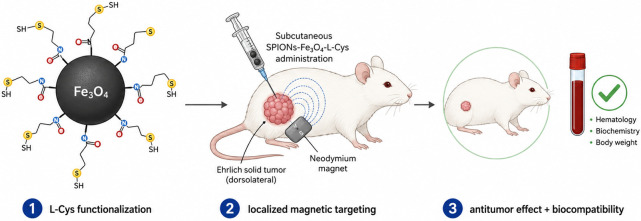

## 1 Introduction

Studying the fundamental pathophysiological processes underlying cancer development necessitates the use of animal models.^1,2^

Mice are the most frequently used animals in biomedical research owing to their small size, ease of handling, adaptability, and high reproductive rate. Their genetic homogeneity also facilitates reproducibility in tumor induction models, such as the Ehrlich carcinoma.^1,3,4^

Ehrlich tumor is a transplantable experimental malignancy of epithelial origin, homologous to mammary adenocarcinoma in female mice. It can develop in different strains, either in an ascitic form when inoculated intraperitoneally or in a solid form when inoculated subcutaneously. A pseudocapsule surrounds the solid Ehrlich tumor and consists of pleomorphic epithelial cells with abundant cytoplasm, sometimes containing subcytoplasmic vacuoles.^2,5,6^

Currently, numerous studies in biomedicine focus on nanotechnology. Functionalized nanoparticles coated with biocompatible materials have attracted increasing interest because they may improve aqueous dispersion, surface reactivity, magnetic responsiveness, and biological interactions.^7,8^ Superparamagnetic iron oxide nanoparticles (SPIONs), such as magnetite (Fe_3_O_4_), possess a size-dependent magnetic behavior characterized by the absence of residual magnetization after the removal of the external magnetic field, which makes them relevant for imaging, targeting approaches, and multifunctional nanosystems.^9,10,11,12^

l-Cysteine is a nontoxic, water-dispersible amino acid with known antioxidant properties. When used as a surface ligand, it may improve nanoparticle dispersion behavior and expose thiol-containing surface chemistry with potential redox-associated biological relevance. In our previous report on the same l-cysteine-functionalized magnetite system, the ligand was shown to be associated with the nanoparticle surface through carboxylate-mediated interactions, while free thiol groups remained detectable in aqueous dispersion by DTNB titration. The same study also showed nanoscale hydrodynamic size, low polydispersity, negative zeta potential, and preserved superparamagnetic behavior, supporting the investigation of this formulation as a biocompatible magnetically responsive nanosystem.^9,13,15^ ^,–^

Cancer cells experience greater oxidative stress due to reactive oxygen species (ROS) compared with normal cells, primarily resulting from genetic alterations and abnormal growth. Consequently, maintaining intracellular glutathione (GSH) is essential for their survival and proliferation in a homeostatic system.^16^ Under high ROS conditions, endogenous l-cysteine (l-Cys) production is insufficient for GSH synthesis, requiring the uptake of extracellular cystine (CSSC) through the xCT(−) transporter.^17^

In cancer patients, the antioxidant defense system is frequently compromised, leading to an accumulation of ROS. This imbalance between free radical production and neutralization is defined as oxidative stress, a condition that promotes tumor growth and disease progression.^18,19^

Considering that l-cysteine may modulate surface charge, aqueous dispersibility, and redox-associated behavior, its functionalization was investigated here as a strategy to obtain a biocompatible, magnetically responsive nanosystem for *in vivo* evaluation. The physicochemical behavior of this formulation, including the presence of free thiol groups in aqueous dispersion and its preserved magnetic response, is further detailed in the Supporting Information.

## 2 Materials and Methods

### 2.1 Synthesis, Functionalization of Nanoparticles, and Cell Toxicity Evaluation

SPIONs-Fe_3_O_4_ were synthesized by coprecipitation of ferric and ferrous salts (2:1 molar ratio) using ammonium hydroxide as the precipitating agent, as previously described. After magnetic separation and washing, the nanoparticles were functionalized with l-cysteine (l-Cys) at mass ratios ranging from 2:1 to 10:1 (l-Cys:Fe_3_O_4_) under vigorous stirring in an aqueous medium for 1 to 5 h. The formulation selected for the present *in vivo* study corresponds to the same l-cysteine-functionalized magnetite system previously characterized in detail by diffraction, vibrational spectroscopy, DTNB thiol quantification, dynamic light scattering, zeta potential, magnetic measurements, and complementary *in vitro* assays. In that previous study, the selected formulation exhibited an average hydrodynamic size of approximately 80 nm, a polydispersity index of 0.22, a zeta potential of about −30 mV, and detectable free thiol groups in aqueous dispersion. In the present work, a localized magnetic field generated by a subcutaneously implanted neodymium magnet was used to establish a magnetic field exposure condition during treatment administration.^9^

Complementary physicochemical characterization of this formulation, including X-ray diffraction, FTIR, surface thiol quantification, magnetic measurements, and complementary *in vitro* data, is provided in the (Supporting Information Figures S1–S13). The same formulation was applied in the *in vivo* study to evaluate biological activity under an external magnetic field generated by a neodymium magnet.^9^ This procedure was used to establish a magnetic-targeting condition during treatment administration.

### 2.2 Animal Model and Experimental Design

Sixty BALB/c mice (30 males, 30 females; 25 ± 5 g) were allocated to experimental groups (n = 6) and maintained under controlled temperature and humidity. The solid Ehrlich tumor was induced by subcutaneous injection of 2 × 10^5^ Ehrlich cells/mL. Treatments started 14 days after inoculation.^2^ The study was approved by CEUA-FMABC (protocol 02/2020) and conducted according to ARRIVE 2.0 guidelines.^14^

Animals were randomly assigned to groups according to treatment and exposure to an external magnetic field. The groups included equal numbers of males and females. Block randomization was generated electronically (Random.org) and balanced for sex and body weight. To reduce bias, the researcher administering treatments did not participate in hematological/biochemical analyses, which were performed by evaluators blinded to group identity; samples were coded before laboratory processing.^20,21^ The design aimed to evaluate the safety, biological effects, and systemic exposure of SPIONs-Fe_3_O_4_-l-Cys previously characterized *in vitro*. Table 1 details the groups, doses, and experimental conditions.

**Table 1:** Experimental Group Allocation of BALB/c Mice, Specifying Treatment, Dosage, Route of Administration, and Exposure to a Magnetic Field

Group	Route	Magnetic Implant	Compound	Dose
**Controls**				
G1 – CTRL (Negative control – baseline)	SC	No	Sodium chloride	0.9%
G2 – MI (Sham: surgery + magnetic implant)	SC	Yes	Sodium chloride	0.9%
G3 – DXR (Positive control – chemotherapy)	IP	No	Doxorubicin	2.5 mg/kg (5 days)
**Treatments without magnetic targeting**				
G4 – Cys100	SC	No	l-Cysteine	100 mg/kg
G5 – NPM	SC	No	SPIONs–Fe_3_O_4_	0.1 mg/kg
G7 – NPMCys50	SC	No	SPIONs–Fe_3_O_4_ + l-Cysteine	0.1 mg/kg/50 mg/kg
G9 – NPMCys100	SC	No	SPIONs–Fe_3_O_4_ + l-Cysteine	0.1 mg/kg/100 mg/kg
**Treatments with magnetic targeting**				
G6 – NPMI	SC	Yes	SPIONs–Fe_3_O_4_	0.1 mg/kg
G8 – NPMCys50MI	SC	Yes	SPIONs–Fe_3_O_4_ + l-Cysteine	0.1 mg/kg/50 mg/kg
G10 – NPMCys100MI	SC	Yes	SPIONs–Fe_3_O_4_ + l-Cysteine	0.1 mg/kg/100 mg/kg

IP, intraperitoneal; SC, subcutaneous; SPIONs, superparamagnetic iron oxide nanoparticles; and MI, magnetic implant. The MI group was considered the sham control, as animals underwent surgical implantation of a neodymium magnet without nanoparticle or drug administration, allowing r the isolation of the effects of surgical manipulation and magnetic exposure. The DXR group (doxorubicin) was included as a positive control for comparative evaluation of antitumor efficacy and systemic safety, as this chemotherapeutic agent has well-established toxicity and efficacy profiles in preclinical models.

### 2.3 Magnet Implantation

Selected groups were anesthetized with isoflurane 2% in O_2_ and underwent subcutaneous implantation of a 2 mm neodymium magnet in the dorsal region, as previously described. This procedure was used to generate a localized magnetic field condition during treatment administration. Because magnetic field strength at the tumor site and nanoparticle biodistribution were not quantified in the present study, this condition should be interpreted as localized magnetic field exposure rather than direct proof of intratumoral magnetic accumulation.^20^ The incision was closed with tissue adhesive. No postoperative analgesia was administered due to the minimally invasive nature of the procedure and rapid wound healing, in line with recommendations for low-impact interventions, provided rigorous welfare monitoring.^22^ Treatments were administered via the IP and SC routes, as specified for each group.

### 2.4 Animal Welfare and Sample Collection

Welfare was monitored daily using a clinical score (0–5 per parameter).^23^ A total score >15 defined the humane end point; euthanasia was performed by anesthetic overdose. Blood was collected by puncture of the caudal vena cava into EDTA and clot-activator tubes for hematology and biochemistry.^24^

### 2.5 Biochemical and Hematological Analyses

Serum assays included iron, total protein, albumin, globulins, urea, creatinine, AST, ALT, ALP, and LDH, using an automated Cobas 8000 (Roche) analyzer. Hematology was performed by flow cytometry on a Sysmex XN-1000, with complete blood counts and smear confirmation.^2,23,25^

### 2.6 Statistical Analysis

Analyses were performed in GraphPad Prism 9.0.1. EC_50_ values were calculated with Quest Graph. One-way ANOVA with Dunnett’s post hoc test, Kruskal–Wallis test, Tukey’s test, and chi-square test were applied as appropriate, with α = 0.05. Data are reported as mean ± SD or median and percentiles according to normality (Shapiro–Wilk). Outliers were identified using the ROUT method (Q = 1%).^26^ Reporting follows ARRIVE 2.0 and best practices in statistical reporting.^21^

## 3 Results

### 3.1 Functionalization with L-Cysteine: *In Vitro* Evidence Supporting *In Vivo* Application

The formulation used in this study was previously characterized in terms of structure, morphology, magnetic properties, colloidal stability, and antioxidant activity in cell-based assays, as described by Lee et al.^19^ SPIONs-Fe_3_O_4_ obtained by the coprecipitation method and functionalized with l-cysteine (l-Cys) exhibited physicochemical properties suitable for biomedical applications, including an average hydrodynamic size of approximately 80 nm, a polydispersity index of 0.22, and a zeta potential of about −30 mV, indicating nanoscale dispersion with low polydispersity and electrostatic stabilization in the aqueous medium. Functionalization with l-cysteine occurred preferentially through carboxylate-mediated association with the nanoparticle surface, while free thiol groups remained detectable in aqueous dispersion by DTNB titration in the previously characterized system. This surface chemistry is consistent with the redox-associated biological activity observed in complementary in vitro assays, although the present in vivo study does not directly quantify thiol persistence after exposure to biological media.^27^

*In* *vitro assays using human mononuclear cells and Ehrlich tumor cell lines* demonstrated a profile of low cytotoxicity and effective antioxidant activity, particularly under oxidative stress conditions induced by nutritional depletion. The mean effective concentration (EC_50_) values for the l-Cys-Fe_3_O_4_ compound were 230.2 μg/mL in unstressed mononuclear cells and 130.1 μg/mL in stressed cells, indicating greater sensitivity to the compound in an oxidative environment. In comparison, nonfunctionalized Fe_3_O_4_ nanoparticles showed significantly lower EC_50_ values (30.7 μg/mL and 77.7 μg/mL, respectively), indicating higher toxicity. In contrast, free l-Cys exhibited intermediate values (74.3 μg/mL and 72.5 μg/mL), supporting the hypothesis that conjugation with the magnetic core increases selectivity and reduces deleterious effects.^27^

These data corroborate previous findings showing that thiol-containing surface functionalization can modify nanoparticle interfacial behavior and redox-related biological responses. In the present context, these effects should be interpreted as biologically relevant surface modulation rather than as evidence of selective cellular targeting.^28^ The low toxicity observed in assays with normal cells suggests that the compound presents a safe application profile, particularly at concentrations between 0.1 and 50 μg/mL, with a wide therapeutic margin. Thus, the *in vitro* data support the proposed application of the l-Cys-Fe_3_O_4_ compound in animal models, allowing advancement to *in vivo* investigations based on preclinical safety and efficacy evidence.^27^

The animal study aimed to evaluate the in vivo biocompatibility, systemic safety, and biological effects of SPIONs-Fe_3_O_4_-l-Cys in a murine model.^27^

According to our previous characterization study of the same l-cysteine-functionalized magnetite system, free thiol groups were quantitatively detected in an aqueous dispersion by DTNB titration, while diffraction and spectroscopic data supported carboxylate-mediated surface association and preservation of the magnetite core. The selected formulation also exhibited a hydrodynamic size of around 80 nm, low polydispersity, negative ζ-potential, and preserved superparamagnetic behavior at 300 K. These data provide physicochemical support for the biological evaluation performed here, although they do not replace the direct assessment of nanoparticle behavior in protein-containing or physiological media.

Furthermore, the formulation used in the present study had previously exhibited an average hydrodynamic diameter of approximately 80 nm, a polydispersity index (PDI) of 0.22, and a zeta potential of approximately −30 mV, indicating a relatively homogeneous nanosuspension with good colloidal stability and electrostatic stabilization in an aqueous medium.^9^

### 3.2 Hematological Profile and Systemic Safety

The hematological evaluation (Table 2) of the animals was performed to assess possible myelotoxic effects resulting from the administration of SPIONs-Fe_3_O_4_ with l-Cys in the different experimental groups. The mean hemoglobin values showed no statistically significant differences between the control group (CTRL) and the treated groups (p = 0.4159), indicating that none of the compounds, including the isolated or functionalized nanoparticles, induced anemia, as observed by Paik et al. (2015),^29^ who evaluated the cytotoxicity of iron oxide nanoparticles in murine bone marrow cells. The results indicated that, at concentrations below 1% (w/v), none of the samples were cytotoxic to bone marrow cells, nor did they affect the expression of cell surface markers or the lipopolysaccharide (LPS)-induced cytokine secretion by murine bone marrow-derived dendritic cells.^28^

**Table 2:** Hematological Parameters of BALB/c Mice across Experimental Groups^a^

Parameters	CTRL	MI	DXR	Cys100	NPM	NPMI	NPMCys50	NPMCys50MI	NPMCys100	NPMCys100MI
RBC (10^6^/μL)	5.02 ± 0.63	5.07 ± 0.49	5.32 ± 0.82	4.58 ± 0.72	4.24 ± 1.06	5.12 ± 0.87	5.66 ± 0.72	4.54 ± 0.89	4.10 ± 0.70	4.68 ± 1.03
HB (g/dL)	15.40 ± 1.00	15.07 ± 0.49	15.32 ± 0.82	14.58 ± 0.72	13.75 ± 1.53	15.36 ± 0.66	15.66 ± 0.72**	14.54 ± 0.89	14.10 ± 0.70**	15.02 ± 0.39
HCT (%)	55.18 ± 1.36	54.82 ± 3.32	53.05 ± 3.86	54.31 ± 2.03	53.41 ± 0.91	51.36 ± 1.61	47.80 ± 3.61	50.70 ± 2.94	47.37 ± 4.78	51.50 ± 2.92
WBC (10^3^/μL)	6.97 ± 1.50	8.40 ± 1.25	10.35 ± 0.99*	4.60 ± 2.68	13.50 ± 1.22	6.43 ± 1.33****	7.31 ± 1.81	6.68 ± 1.37	5.91 ± 1.40	4.52 ± 1.30
NEU (10^3^/μL)	1.08 ± 0.26	1.45 ± 0.59	1.64 ± 1.31	0.34 ± 0.24	0.28 ± 0.09	2.71 ± 0.89***	1.36 ± 0.75	1.07 ± 0.26	0.73 ± 0.10	0.9 ± 0.55
LIN (10^3^/μL)	7.33 ± 0.56	7.05 ± 0.92	8.23 ± 1.02	5.62 ± 1.32	5.20 ± 1.17	7.63 ± 1.85	5.61 ± 2.03	5.69 ± 0.95	3.50 ± 0.55***	5.13 ± 1.55
MON (10^3^/μL)	0.42 ± 0.42	0.36 ± 0.18	0.62 ± 0.39	0.31 ± 0.37	0.11 ± 0.11	0.52 ± 0.39	0.09 ± 0.12	0.38 ± 0.46	0.54 ± 0.10	0.33 ± 0.20
PLT (10^3^/μL)	421.60 ± 75.13	1154.75 ± 93.68****	1121.50 ± 84.60****	429.42 ± 79.32	598.16 ± 37.26**	1269.20 ± 82.77****	551.83 ± 42.22	802.25 ± 112.91****	574.66 ± 71.14	831.33 ± 79.01****
RNL	0.25 ± 0.12	0.20 ± 0.07	0.25 ± 0.12	0.05 ± 0.05	0.05 ± 0.02	0.38 ± 0.13	0.22 ± 0.15	0.33 ± 0.25	0.07 ± 0.04	0.24 ± 0.13

a RBC (10^6^/μL): Red blood cells per microliter of blood; HCT (%): Hematocrit; HB (g/dL): Hemoglobin in grams per deciliter of blood; WBC (10^3^/μL): White blood cells per microliter of blood; NEU (10^3^/μL): Neutrophils per microliter of blood; LIN (10^3^/μL): Lymphocytes per microliter of blood; MON (10^3^/μL): Monocytes per microliter of blood; PLT­(103/μL): Platelets per microliter of blood; RNL: Neutrophil/lymphocyte ratio. *, **, *** and **** in a cell indicate that a group is significantly different compared to the control group (Dunnett’s multiple comparison).

The group treated with Doxorubicin (DXR), despite its well-recognized hematological toxicity,^30^ did not show significant alterations in hemoglobin levels (p ≥ 0.0999), possibly due to the short exposure period or the administered dose.^31^ The other groups treated with SPIONs-Fe_3_O_4_ anchored with l-cysteine at both concentrations maintained stable levels, demonstrating the absence of relevant toxic effects on hemoglobin synthesis.

The red blood cell count remained within the physiological values for the species and without statistically significant differences (p = 0.121), with a slight reduction observed in the groups treated with NPM and NPMCys100, with no associated clinical significance. On the other hand, the NPMCys50MI group showed a slight increase in erythrocyte count, possibly related to a stimulatory and protective effect on erythropoiesis,^32,33^ suggesting an influence of l-Cys as an antioxidant agent.^34^

Hematocrit values followed the same pattern as that of hemoglobin and red blood cell counts, confirming the integrity of the erythroid series. The NPMCys50MI (p = 0.0016) and NPMCys100MI (p = 0.0037) groups showed small reductions, still within physiological limits, not suggesting any possible myelotoxicity.^32^

The leukogram demonstrated a significant increase in total leukocyte count in the DXR (p = 0.0255) and NPMI (p ≤ 0.0001) groups, suggesting moderate leukocytosis that may reflect a compensatory immune response to drug toxicity. However, in other contexts, DXR can cause myelosuppression^33^ depending on the dose and exposure time.

Marked leukocytosis was also observed with the administration of SPIONs-Fe_3_O_4_ with a magnetic implant (NPMI) at the administered dose, as reported by Shah and Dobrovolskaia (2019), which highlighted that nanoparticles can interact with different types of immune cells, such as monocytes and neutrophils, potentially leading to changes in blood cell counts and modulation of immune system activity.^35^ Another hypothesis suggests that the NPMI group presented marked leukocytosis, possibly related to the local inflammatory response triggered by the metallic implant.^33^ This indication is reinforced by the significant increase in neutrophil count (p = 0.0002), consistent with an early inflammatory response. These findings point to a possible modulatory effect on inflammation and support the hypothesis of an acute local inflammatory process.^33,35^

The peripheral blood lymphocyte count is important for assessing the impact of treatments on adaptive immunity. It was observed that the NPMCys100MI group showed significant reductions compared to the CTRL group (p = 0.0001); this finding indicates a possible immunosuppressive effect or lymphocyte redistribution induced by the interaction between the compound and the inflammatory microenvironment.^36^

Regarding monocytes, no statistically significant differences were observed between treated groups (p = 0.1988), even when compared to the control group, indicating the stability of this immunological parameter across treatments.

The significant increase in platelet count in the DXR group suggests a systemic inflammatory response, common in situations of tissue damage or oxidative stress induced by toxic agents such as doxorubicin, which can lead to secondary reactive thrombocytosis through systemic stress mediated by cytokines such as IL-6 and growth factors such as thrombopoietin, thereby stimulating platelet production.^37^ Similarly, groups that received the magnetic implant also showed increased platelet counts, with statistically significant differences compared to CTRL in the MI (p < 0.0001), NPMI (p < 0.0001), NPMCys50MI (p < 0.0001), and NPMCys100MI (p < 0.0001) groups, which may indicate an inflammatory response due to tissue trauma associated with the implant.^38^ Such a response may stimulate the production of inflammatory mediators such as IL-6 and TNF-α, which are recognized inducers of thrombopoiesis.^37,38,39^ This finding suggests that the magnetic implant, although potentially useful in the therapeutic targeting of nanoparticles, may also contribute to relevant hematological alterations, requiring careful long-term biocompatibility assessment.

The neutrophil-to-lymphocyte ratio (NLR) has been widely used as a prognostic marker in various clinical conditions and preclinical models, reflecting the balance between the innate immune response (neutrophils) and the adaptive response (lymphocytes). Elevated NLR values are associated with a worse prognosis in inflammatory, neoplastic, and infectious diseases. Their increase is considered indicative of immunological stress, systemic inflammation, or lymphocytic immunosuppression.^40,41^ In the present study, the groups that received the magnetic implant showed higher NLR values, with differences among groups (p = 0.0116), but without statistical significance when compared to the control group, thus suggesting the presence of an inflammatory condition^39,42^ possibly due to surgical manipulation and the metallic implant.

### 3.3 Biochemical Parameters and Organ Function

The biochemical evaluation (Table 3) aimed to identify potential metabolic, inflammatory, renal, and hepatic alterations associated with treatment with l-cysteine–functionalized SPIONs-Fe_3_O_4_ (SPIONs-Fe_3_O_4_-l-Cys), in comparison with the control groups.

**Table 3:** Serum Biochemical Parameters of the Different Experimental Groups^a^

Parameters	CTRL	MI	DXR	Cys100	NPM	NPMI	NPMCys50	NPMCys50MI	NPMCys100	NPMCys100MI
FeS (ug/dL)	143.20 ± 143.20	273.05 ± 59.45	171.60 ± 29.61	156.57 ± 24.07	640.66 ± 156.90****	516.33 ± 67.71****	448.14 ± 41.55****	211.60 ± 40.16	455.85 ± 50.96****	208.28 ± 27.57
PT (mg/dL)	5.47 ± 5.47	5.00 ± 0.09	4.26 ± 0.33**	4.76 ± 0.29	4.78 ± 0.39	5.75 ± 0.81***	4.20 ± 0.51*	4.42 ± 0.32**	4.51 ± 0.20	4.44 ± 0.35**
ALB (mg/dL)	2.52 ± 2.52	2.38 ± 0.28	0.78 ± 0.34***	2.36 ± 0.45	3.24 ± 0.78	2.46 ± 0.39	1.35 ± 0.67**	1.77 ± 0.46	1.09 ± 0.44***	1.75 ± 0.72
GLB (mg/dL)	2.75 ± 0.3	2.62 ± 0.27	3.36 ± 0.40	2.18 ± 0.36	2.10 ± 0.57	2.04 ± 0.65	2.72 ± 0.81	2.61 ± 1.13	4.00 ± 0.86	2.98 ± 0.98
CRE (mg/dL)	0.14 ± 0.05	0.18 ± 0.04	0.13 ± 0.05	0.18 ± 0.08	0.14 ± 0.09	0.21 ± 0.08	0.07 ± 0.05	0.14 ± 0.09	0.11 ± 0.08	0.17 ± 0.17
URE (mg/dL)	45.82 ± 7.66	57.62 ± 9.07	47.80±6.49	50.38 ± 9.45	40.41 ± 4.46	44.10 ± 4.52	46.98 ± 11.31	53.90 ± 9.51	49.68 ± 6.03	44.38 ± 4.40
AST (U/L)	224.00 ± 60.10	736.83 ± 42.83****	486.75 ± 36.47***	225.00 ± 37.04	370.28 ± 75.89*	695.60 ± 86.63****	282.25 ± 93.20	561.00 ± 102.91****	182.66 ± 39.06	611.66 ± 91.23****
ALT (U/L)	52.88 ± 11.15	43.80 ± 16.63	134.50 ± 26.93****	48.16 ± 10.45	69.40 ± 19.75	55.33 ± 14.97	44.50 ± 18.26	26.83 ± 3.71	46.50 ± 25.95	60.66 ± 21.71
ALP (U/L)	31.66 ± 8.21	34.50 ± 5.89	72.66 ± 9.43****	30.83 ± 2.13	34.66 ± 3.44	58.00 ± 14.35****	26.66 ± 6.56	38.00 ± 1.78	32.50 ± 6.05	24.33 ± 2.73
LDH (U/L)	4879.25 ± 1298.58	4452.40 ± 1120.66	4498.66 ± 1307.64	4008.83 ± 1141.94	3878.14 ± 516.94	5145.66 ± 1417.95	3172.33 ± 368.22	2082.00 ± 642.00**	2668.00 ± 1952.73*	2352.12 ± 526.73**

a Data are presented as mean value ± standard deviation. FeS (μg/dL): Serum Iron, micrograms per deciliter; PT (mg/dL): Total protein, milligrams per deciliter; ALB (mg/dL): Albumin, milligrams per deciliter; GLB (mg/dL): Globulin, milligrams per deciliter; CRE (mg/dL): Creatinine, milligrams per deciliter; URE (mg/dL): Urea, milligrams per deciliter; AST (U/L): Aspartate aminotransferase, units per liter; ALT (U/L): Alanine aminotransferase, units per liter; ALP (U/L): Alkaline phosphatase, units per liter; LDH (U/L): Lactate dehydrogenase, units per liter. *, **, *** and **** in a cell indicate that a group is significantly different compared to the control group (Dunnett’s multiple comparison).

In the analysis of serum iron levels among the different groups, important alterations were evidenced regarding iron bioavailability associated with l-Cys–functionalized SPIONs. A significant increase was observed in the groups administered SPIONs-Fe_3_O_4_ associated with l-Cys and in the other groups without a magnetic implant, reaching statistical significance in the MI (p = 0.0265), NPM (p < 0.0001), NPMI (p < 0.0001), NPMCys50 (p < 0.0001), and NPMCys100 (p < 0.0001) groups compared to the control group, with NPMI showing the highest elevations (516.3 ± 67.7 μg/dL).

Serum iron levels varied significantly among the groups. The NPMI (p = 0.0265) and NPMCys100 (p = 0.0265) groups showed elevated iron concentrations compared to those of CTRL, which can be attributed to the controlled release of iron from the nanoparticles. This finding is consistent with the magnetic nature of the tested compounds. The MI group (p = 0.0265) also showed an increased serum iron level, possibly related to oxidative or inflammatory processes. In contrast, Cys100 and DXR maintained lower levels, suggesting a possible interference with iron absorption or transport.

The elevation of iron levels in groups treated with nanoparticles (NPMI and NPMCys100) is consistent with the gradual release of iron from SPIONs, as previously described in biodistribution and metabolism studies. According to the ref,^43^ iron bioavailability can be modulated by factors such as surface functionalization and colloidal stability,^43^ which may explain the differences observed among groups. On the other hand, the reduced levels in the DXR and Cys100 groups suggest impaired absorption or increased tissue uptake, possibly due to systemic inflammatory processes associated with drug-induced toxicity.^44,45^

Total protein concentrations remained relatively stable among groups, with slight increases in the NPM and MI groups. The DXR group showed slightly reduced values (p = 0.00166), which may be related to hepatic alterations due to drug toxicity.^46^

Albumin levels showed a significant reduction in the DXR group (p < 0.0001), indicating possible hepatic impairment, as albumin is synthesized exclusively by the liver.^46^ The NPM group exhibited the highest albumin levels, followed by NPMI, suggesting preserved liver function, as also observed in other studies.^27,29^ The NPMCys group maintained values within the physiological range, indicating good hepatic tolerability.

Although this study did not include specific immunological analyses, some indirect parameters, such as globulin variation and changes in the leukogram, suggest a possible mild activation of the innate response, particularly in groups with magnetic implants and those treated with doxorubicin. In the NPMCys groups, moderate increases in globulins, particularly in NPMCys100, may reflect mild immune stimulation without evidence of exacerbated inflammation. It should be emphasized, however, that these findings must be interpreted with caution, since molecular markers or inflammatory cytokines were not evaluated. In this sense, future studies will be directed toward gene expression analysis of inflammatory markers, which will more robustly elucidate the immunological impact of functionalized SPIONs.^47,48,49^

The stability of total protein levels indicates the preservation of overall hepatic integrity. The reduction in albumin in the DXR and Cys100 groups reinforces the hepatotoxic profile of these agents, since albumin synthesis is highly sensitive to early parenchymal injury. Studies, such as ref^50^ have demonstrated that doxorubicin reduces the expression of genes related to hepatic protein synthesis.^50^

Serum creatinine levels remained within physiological limits in all groups, with slight variations of no clinical significance. The NPMI group showed a slight increase but without statistical significance; groups treated with NPMCys did not show significant alterations at any dose, evidencing the absence of nephrotoxicity.^51^ Regarding serum urea, no biochemical alterations or statistical correlations were observed, indicating that none of the compounds induced protein catabolism beyond the physiological threshold or led to renal impairment.^51^

A pattern of alteration in hepatic markers was observed in this study, particularly alanine aminotransferase (ALT), a sensitive marker of parenchymal liver injury, especially in cases of drug-induced hepatotoxicity.^52^ The DXR group showed a significant increase (p < 0.0001), nearly doubling control group values, evidencing the hepatotoxic behavior of doxorubicin, as observed in other studies;^47,53^ in the other groups, values remained within the normal range.

The increase in serum aspartate aminotransferase (AST) observed in animals submitted to surgery for magnetic implantation (MI, NPMI, NPMCys50MI, and NPMCys100MI) may be associated with the physiological response to tissue trauma and local inflammation resulting from the procedure. AST is an enzyme widely distributed in tissues such as the liver, skeletal muscle, and myocardium, and its release is common in situations of surgical stress and muscle injury.^52^ Additionally, the presence of the implant may induce an acute or subacute inflammatory response, leading to altered cell integrity and the release of enzymes into the circulation. Previous studies have also suggested that magnetic materials, even when biocompatible, may promote transient changes in biochemical profiles, especially if interacting with metabolically active tissues or through the controlled release of metallic ions, such as iron, in the microenvironment.^54^ These findings reinforce the need for integrated evaluation with other hepatic and muscular markers to distinguish the origin of the enzymatic alteration.^14,45^

The groups treated with DXR (p < 0.0001) and NPM (p < 0.0001) showed significant increases in alkaline phosphatase levels, corroborating the hepatic findings observed in other biochemical markers. In the other groups, values remained within the normal range, indicating the absence of relevant cholestasis hepatic alterations.

Animals from the NPMCys50MI (p = 0.0018) and NPMCys100MI (p = 0.0032) groups showed significantly lower lactate dehydrogenase (LDH) levels compared to the CTRL group, indicating lower tumor cell aggressiveness and greater antioxidant potential due to l-Cys anchoring. These results are consistent with recent approaches that use nanoplatforms to modulate the hypoxic tumor microenvironment.^55,56^ The MI (4452.4 ± 1120.7 U/L) and DXR (4498.7 ± 1307.6 U/L) groups showed the highest concentrations, consistent with greater cellular activity, tumor necrosis, and possible systemic toxicity due to doxorubicin use.^57,58^ These findings suggest that, in addition to the tumor’s role as an inducer of tissue injury, therapeutic approaches based on functionalized nanoparticles may also exert a modulatory effect, partially protecting against the exacerbated release of this cellular damage marker enzyme.^58,59^

### 3.4 Functional and Antitumor Evaluation

Complementary characterization supporting the physicochemical behavior of the formulation is now presented in the Supporting Information, including XRD patterns demonstrating the preservation of the magnetite crystalline phase, FTIR spectra supporting cysteine functionalization, DTNB-based surface thiol quantification, magnetic hysteresis data confirming superparamagnetic behavior, and complementary in vitro cytotoxicity results.

The welfare score, measured through a daily observational scoring system according to the NC3Rs protocol (National Centre for the Replacement, Refinement, and Reduction of Animals in Research),^24^ showed no statistically significant difference among the groups (p = 0.4109), as illustrated in Figure 1. This indicates that, regardless of the treatment applied, including the use of l-Cys–functionalized SPIONs (NPMCys), the animals maintained clinical conditions compatible with preserved welfare, without evidence of significant suffering.

**Figure 1:**
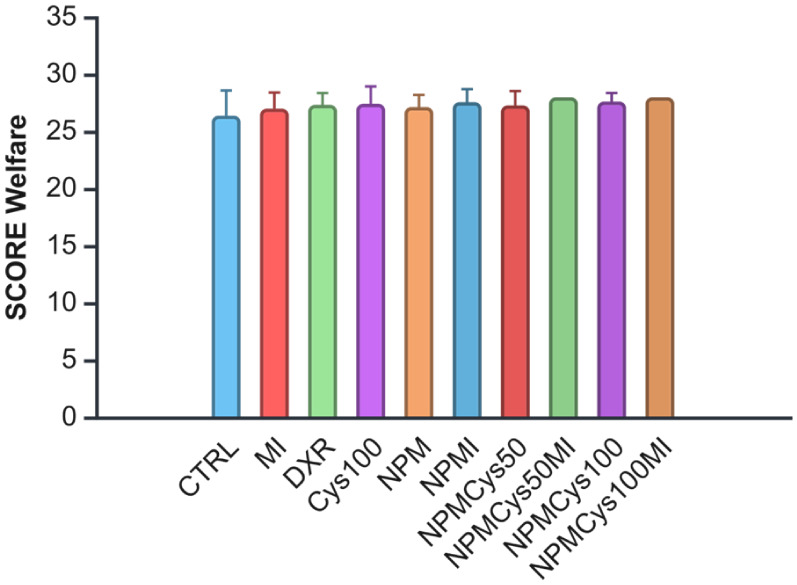
Animal welfare scores assessed throughout the experimental period in mice bearing Ehrlich solid tumors subjected to different treatments. Data represent mean ± standard deviation of the cumulative welfare score for the following groups: CTRL (control), MI (magnetic implant), DXR (doxorubicin), Cys100 (l-cysteine 100 mg/kg), NPM (nonfunctionalized superparamagnetic iron oxide nanoparticles), NPMI (nonfunctionalized nanoparticles with magnetic implant), NPMCys50, NPMCys50MI, NPMCys100, and NPMCys100MI (l-cysteine-functionalized SPIONs at 50 or 100 mg/kg, with or without magnetic targeting). No statistically significant differences were observed among groups (one-way ANOVA, *p* > 0.05), indicating preserved animal welfare across all experimental conditions.

These findings corroborate previous studies attesting to the biocompatibility of SPIONs coated with amino acids such as l-Cys, which is recognized for its low toxicity and antioxidant properties that minimize side effects in murine models.^9,60^

Tumor weight showed statistically significant differences among the experimental groups. It was observed that the groups treated with NPMCys50MI (p < 0.0001) and NPMCys100MI presented the lowest mean tumor masses, particularly the NPMCys50MI group (Figure 2), suggesting attenuation of tumor burden under the experimental conditions adopted for the anchored and magnetically targeted formulations. Because tumor weight is a macroscopic end point, the present interpretation is restricted to gross antitumor response and should not be interpreted as direct evidence of microscopic tissue architecture, residual infiltration, or intratumoral distribution.^61^

**Figure 2:**
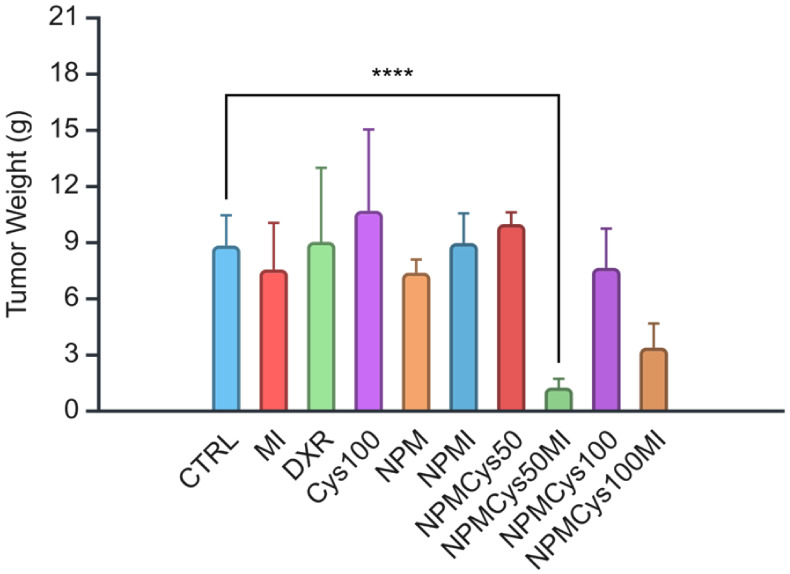
Tumor weight measured at the end of the experimental period in mice bearing Ehrlich solid tumors subjected to different treatments. Data are presented as mean ± standard deviation for the following groups: CTRL (control), MI (magnetic implant), DXR (doxorubicin), Cys100 (l-cysteine 100 mg/kg), NPM (nonfunctionalized superparamagnetic iron oxide nanoparticles), NPMI (nonfunctionalized nanoparticles with magnetic implant), NPMCys50, NPMCys50MI, NPMCys100, and NPMCys100MI (l-cysteine-functionalized SPIONs at 50 or 100 mg/kg, with or without magnetic targeting). A significant reduction in tumor weight was observed in animals treated with l-cysteine-functionalized SPIONs under magnetic targeting, particularly in the NPMCys50MI group (****p < 0.0001; one-way ANOVA followed by appropriate post hoc analysis).

These results are consistent with the biological performance expected for magnetically responsive and biocompatible-functionalized SPION systems. In the present study, the reduction in tumor burden observed under magnetic targeting conditions supports an association between l-cysteine functionalization, preserved magnetic responsiveness, and antitumor effect under the adopted experimental conditions, although direct intratumoral accumulation was not measured.^62,63,64^

In addition, the analysis of tumor size variation by pachymetry (Figure 3) reinforces the findings observed in the tumor mass. In the control (CTRL), MI (p = 0.0019), and NPM (p = 0.0018) groups, there was significant progressive tumor growth. In contrast, the NPMCys50MI and NPMCys100MI groups demonstrated containment of progression, with a tendency toward volumetric regression in some individuals.

**Figure 3:**
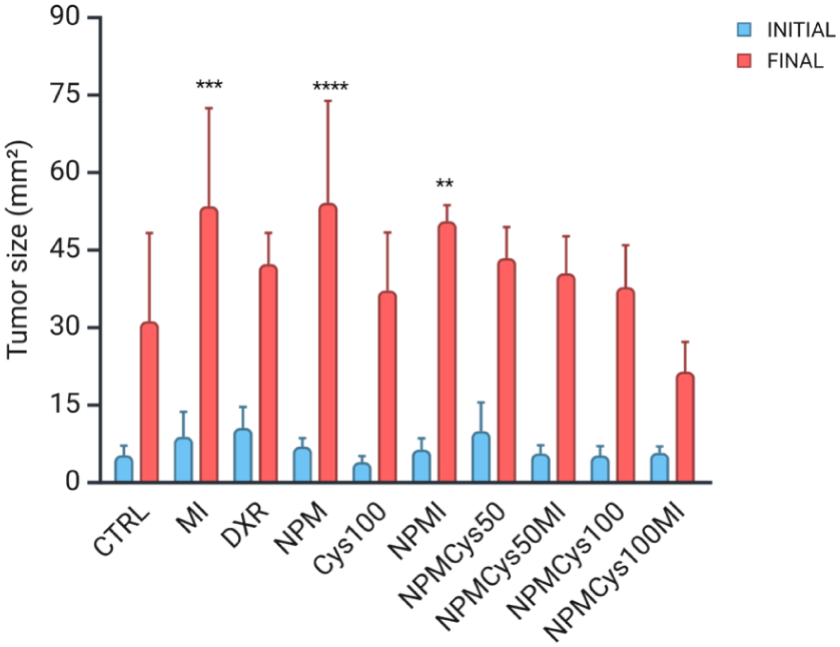
Tumor size progression assessed by pachymetry in mice bearing Ehrlich solid tumors before (initial) and after (final) the experimental treatments. Data are expressed as mean ± standard deviation of tumor area (mm^2^) for the following groups: CTRL (control), MI (magnetic implant), DXR (doxorubicin), Cys100 (l-cysteine 100 mg/kg), NPM (nonfunctionalized superparamagnetic iron oxide nanoparticles), NPMI (nonfunctionalized nanoparticles with magnetic implant), NPMCys50, NPMCys50MI, NPMCys100, and NPMCys100MI (l-cysteine-functionalized SPIONs at 50 or 100 mg/kg, with or without magnetic targeting). Significant increases in tumor size were observed in the MI, DXR, and NPM groups, whereas treatments with l-cysteine-functionalized SPIONs under magnetic targeting attenuated tumor progression, with reduced final tumor dimensions. Statistical significance between initial and final measurements is indicated by **p < 0.01, ***p < 0.001, and ****p < 0.0001.

In Figure 3, it was observed that the variation in tumor diameter in the groups treated with anchored SPIONs was more discrete compared to the others, with tumor volumes remaining practically stable over time. These data suggest attenuation of tumor progression in the groups treated with functionalized magnetic formulations under magnetic targeting conditions, without supporting a direct mechanistic inference regarding tumor accumulation or intracellular delivery.

Previous studies have shown that SPION-based systems may display favorable magnetic responsiveness and biological applicability in experimental models. In the present study, l-cysteine functionalization was associated with preserved magnetic behavior and biocompatibility, while the in vivo findings support antitumor activity under magnetic-targeting conditions. However, the present data set does not allow direct conclusions regarding EPR-mediated internalization, intratumoral biodistribution, or payload delivery.^65^ Anchoring l-Cys to SPIONs promoted a biocompatible, functional, and responsive system to the oxidative tumor microenvironment, favoring targeted delivery and a superior antineoplastic response.^66,67^

The body weight of the animals, used as an indirect indicator of systemic toxicity, showed no statistically significant alterations among the groups (p = 0.4854). The data suggest that the tested formulations, including both isolated and functionalized SPIONs, did not induce relevant adverse effects that compromised nutritional status or caused significant weight loss, reinforcing the safety profile of the nanoparticles. According to the ref,^64^ cysteine-containing formulations show high tolerability in animal models, even after prolonged administration, which is consistent with the findings of the present study.^64^

The present study has important limitations. Although the formulation was supported by prior physicochemical characterization of the same l-cysteine-functionalized magnetite system, we did not directly quantify nanoparticle biodistribution, magnetic field intensity at the tumor site, colloidal stability in protein-containing media, or thiol persistence after biological exposure. Therefore, our findings should be interpreted as evidence of biocompatibility and antitumor-associated effects under a localized magnetic field condition rather than as direct proof of tumor-selective delivery.

## 4 Discussion

The present study demonstrated that superparamagnetic iron oxide nanoparticles functionalized with l-cysteine (NPMCys) exhibit a favorable profile of biocompatibility, low toxicity, and antitumor potential in a murine model of mammary adenocarcinoma induced by an Ehrlich tumor. The formulation preserved relevant physicochemical properties, showed reduced tumor burden— especially when combined with an external magnetic field—and did not induce hematological or biochemical alterations indicative of overt systemic toxicity under the experimental conditions adopted. Preservation of hepatic and renal function, maintenance of animal welfare, and the absence of significant body weight loss reinforce the tolerability of the proposed nanosystem.

It is possible that functionalization with l-cysteine contributed to the biological performance of the formulation through combined redox-related and magnetic effects under the experimental conditions used. However, the present data set does not allow definitive mechanistic conclusions. Magnetic targeting appeared to enhance the observed antitumor response, which supported the relevance of localized magnetic responsiveness in this model.

## Supplementary Material



## Data Availability

The data supporting the findings of this study are included in the Supporting Information accompanying this manuscript and will be made publicly available by the journal upon the publication of the article.

## Notes

X-ray diffraction patterns; FTIR spectra; DTNB-based surface thiol quantification; magnetic measurements; hydrodynamic size, polydispersity index, and zeta potential data; in vitro cytotoxicity and antioxidant assays; and supplementary experimental details (PDF)
