# Supplementary material for: Biocompatibility and Antitumor Effects of Magnetically Targeted L‑Cysteine-Functionalized Superparamagnetic Iron Oxide Nanoparticles in an Ehrlich Tumor Model
Source: ACS Omega. 2026 Jul 17;11(29):43277–87. doi: 10.1021/acsomega.6c01138 (PMC13425479; doi:10.1021/acsomega.6c01138)
Supplement: Supplementary file 1 [file ao6c01138_si_001.pdf]

Supporting Information

Biocompatibility and Antitumor Effects of Magnetically Targeted L-Cysteine-Functionalized Superparamagnetic Iron Oxide Nanoparticles in an Ehrlich Tumor Model

Authors:

Emerson Barbosa da Silva[1*], Tatiane Nassar Britos[3], Camila dos Santos Chagas[1], Giuliana Petri[2], Glaucia Raquel Luciano da Veiga[1], Beatriz da Costa Aguiar Alves Reis[1], Paula Silvia Haddad[3], Fabio Furlan Ferreira[4], Fernando Luiz Affonso Fonseca[1,5]

Affiliations:

1. Department of Pathology, Centro Universitário FMABC (FMABC), Santo André, SP , Brazil
2. Experimental Biotery Laboratory of the FMABC, Centro Universitário FMABC (FMABC), Santo André, SP, Brazil.
3. Department of Chemistry, Federal University of São Paulo (UNIFESP), Diadema, SP, Brazil
4. Center for Natural and Human Science (CCNH), Federal University of ABC (UFABC), Santo André, SP, Brazil
5. Department of Pharmaceutical Sciences, Federal University of São Paulo (UNIFESP), Diadema Campus, Diadema, SP, Brazil

Corresponding author:

Emerson Barbosa da Silva, Department of Pathology, Centro Universitário FMABC, Av. Lauro Gomes 2000, Vila Sacadura Cabral, Santo André, São Paulo, 09060-870, SP, Brazil.
Email: emerson.silva@fmabc.br

Supporting Information for Publication

SUPPLEMENTARY INFORMATION

Biocompatibility and Antitumor Effects of Magnetically Targeted L-Cysteine-Functionalized Superparamagnetic Iron Oxide Nanoparticles in an Ehrlich Tumor Model

S1. Synthesis of Superparamagnetic Iron Oxide Nanoparticles

Superparamagnetic iron oxide nanoparticles (SPIONs) were synthesized using the chemical co-precipitation method. In this procedure, aqueous solutions of ferric chloride ($FeCl_3$, 0.5 mol L^{-1}) and ferrous chloride ($FeCl_2$, 1.0 mol L^{-1}) were mixed in a volumetric ratio of 4:1 and subsequently reacted with ammonium hydroxide (NH_4OH), resulting in the formation of magnetite nanoparticles (Fe_3O_4).

This method was selected because it is simple, reproducible, and capable of producing hydrophilic nanoparticles with adequate aqueous dispersibility for subsequent physicochemical and biological evaluation.

S2. Surface Functionalization with L-Cysteine

The SPIONs were functionalized with L-cysteine (Cys), a hydrophilic amino acid with antioxidant properties. The carboxyl group (–COOH) of cysteine interacts with the iron oxide surface while the thiol groups (–SH) remain exposed.

These thiol groups contribute to the redox-associated properties of the nanosystem and may be relevant for future surface derivatization strategies, although no therapeutic cargo was incorporated in the present study.

S3. X-Ray Diffraction Analysis

X-ray diffraction (XRD) measurements were performed to confirm the crystalline structure of the nanoparticles and to evaluate the influence of surface functionalization on the magnetite structure.

The diffraction peaks correspond to the characteristic crystallographic planes of magnetite (Fe_3O_4). The average crystallite size calculated using the Scherrer equation considering the (311) reflection plane was approximately 12 nm for SPIONs and 13 nm for SPION-Cys nanoparticles, indicating that surface functionalization does not significantly affect the crystalline structure of the magnetic core.

Evidence of cysteine dimerization into cystine in the solid state was also observed through characteristic diffraction peaks associated with cystine crystalline phases.

Figure S1. X-ray diffraction patterns of pure SPIONs and SPION-Cys nanoparticles functionalized at different reaction times (1 h, 3 h, 5 h, and 7 h) using a SPION:Cys mass ratio of 1:10.

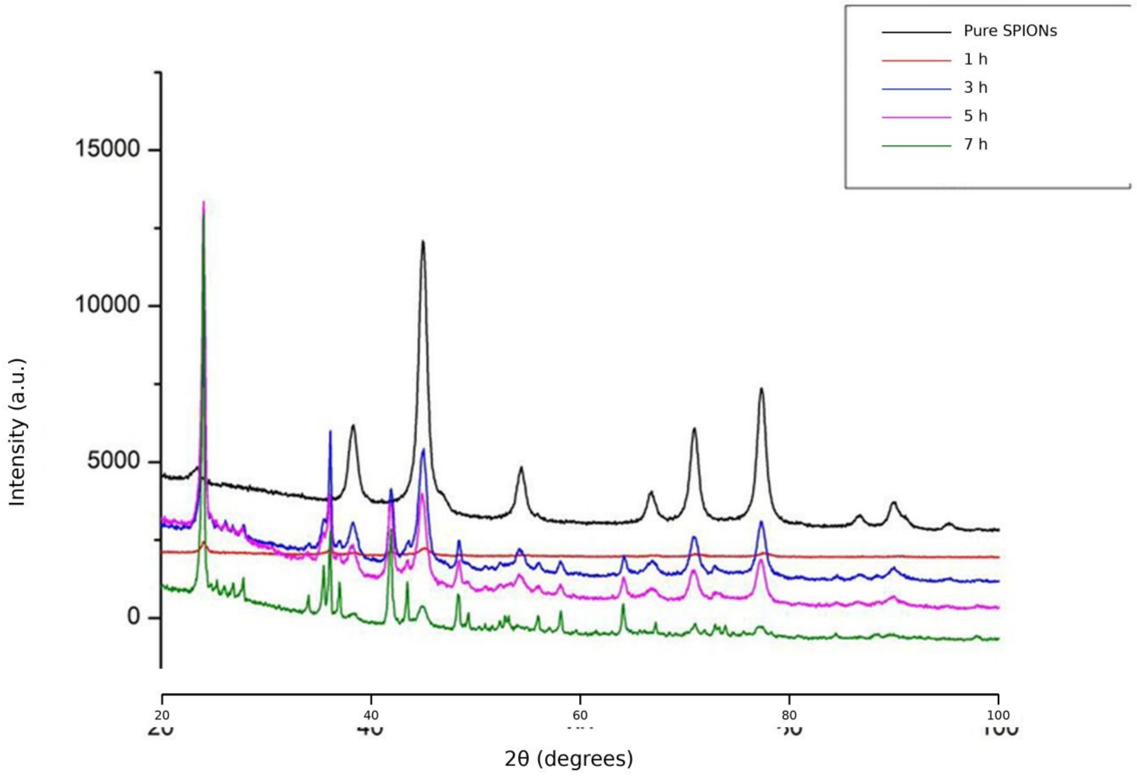

Figure S2. Normalized X-ray diffraction patterns of L-cysteine (black), pure SPIONs (blue), and SPION-Cys nanoparticles (red), showing reflections corresponding to magnetite crystalline structure.

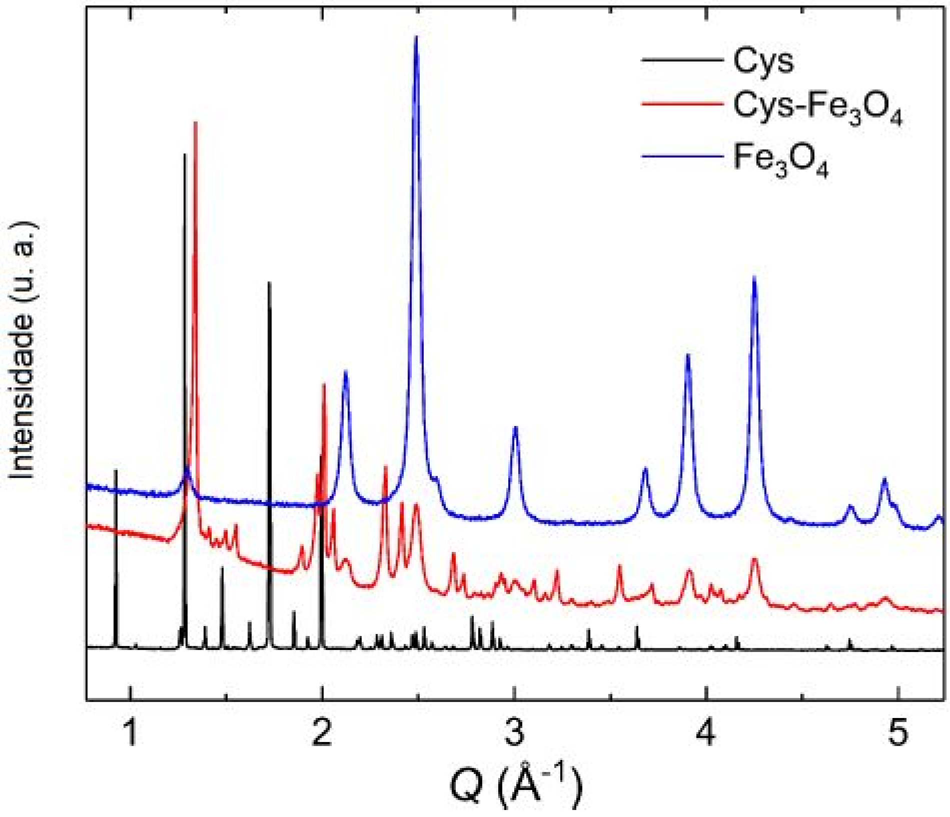

Figure S3. X-ray diffraction patterns of SPION-Cys nanoparticles synthesized for 1 h using different SPION:Cys mass ratios (1:2, 1:4, 1:6, and 1:8).

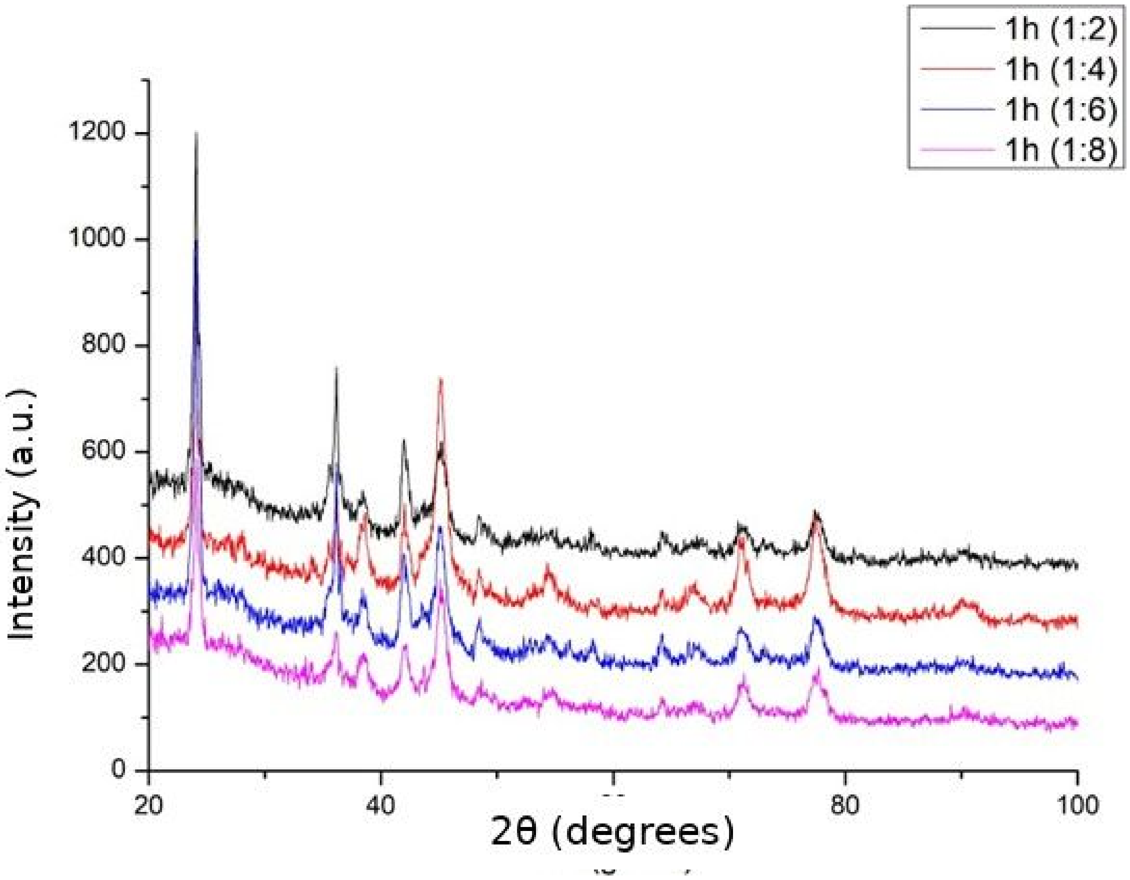

Figure S4. X-ray diffraction patterns of SPION-Cys nanoparticles synthesized for 3 h using different SPION:Cys mass ratios (1:2, 1:4, 1:6, and 1:8).

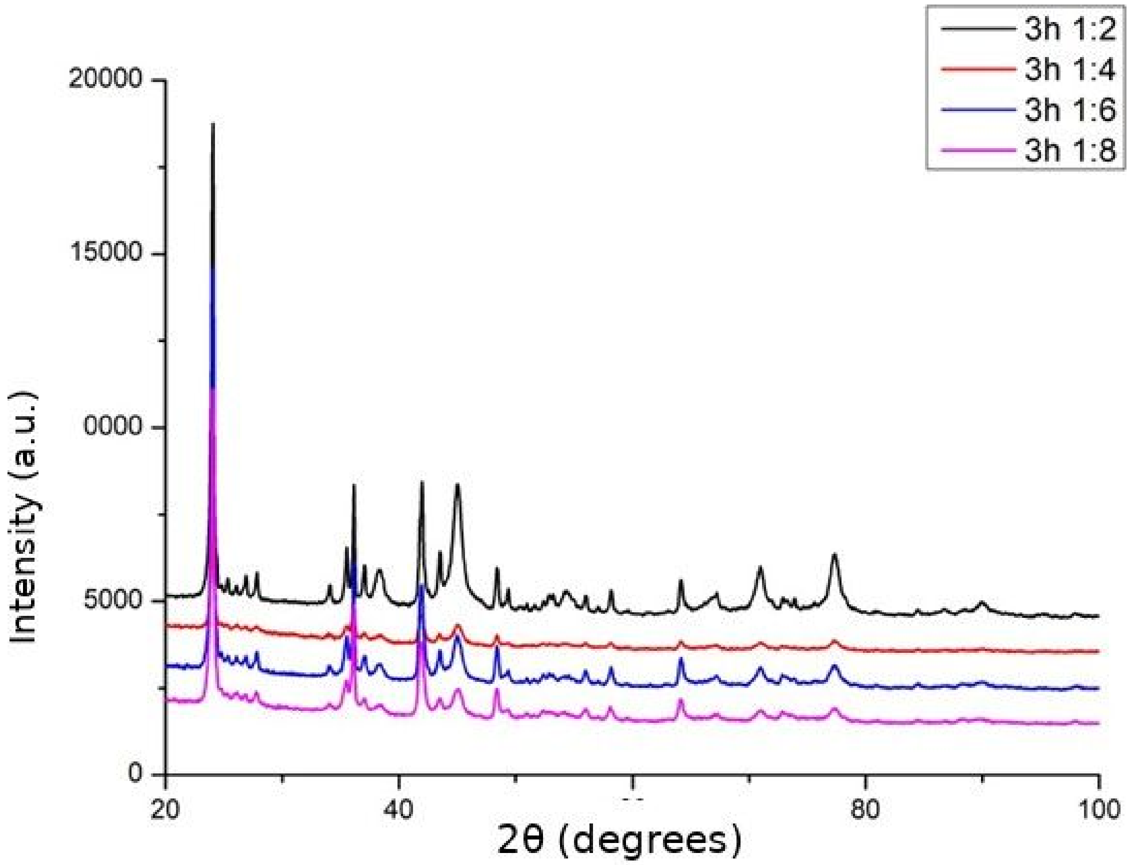

Figure S5. X-ray diffraction patterns of SPION-Cys nanoparticles synthesized for 5 h using different SPION:Cys mass ratios (1:2, 1:4, 1:6, and 1:8).

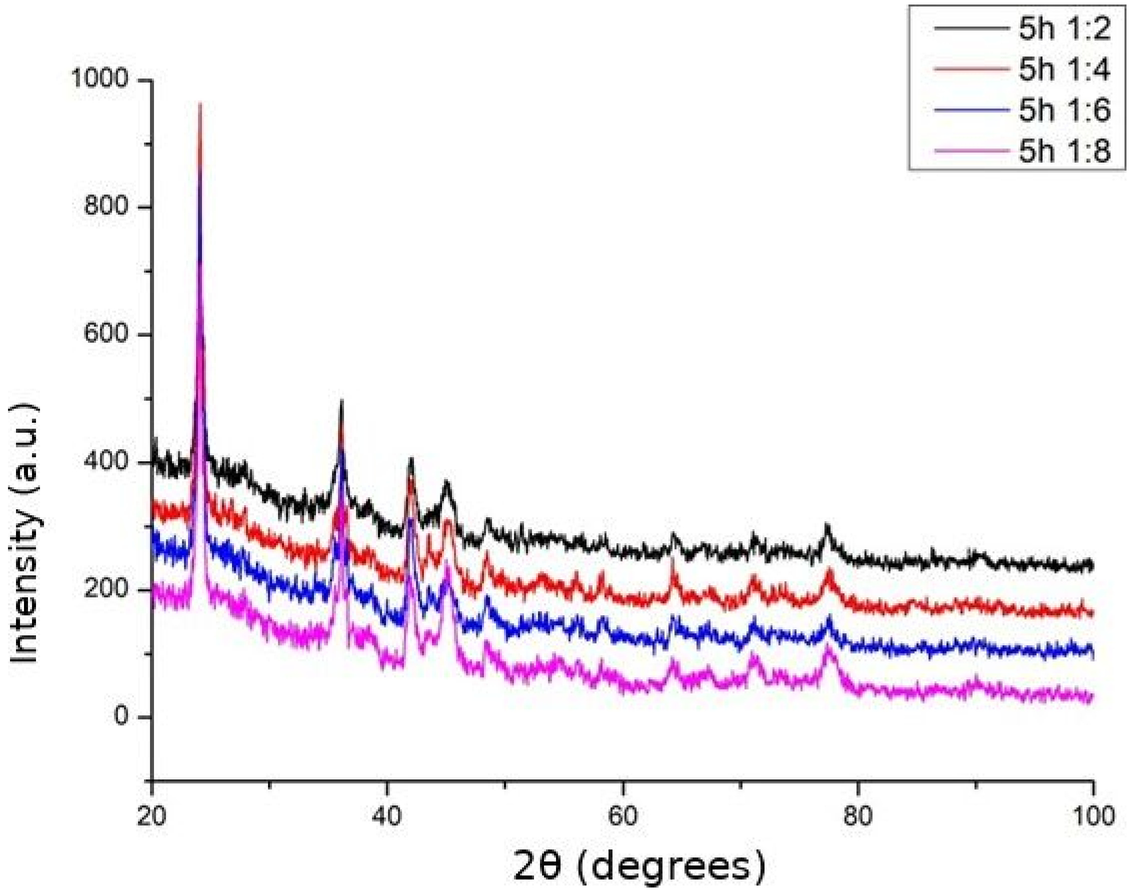

S4. FTIR Spectroscopic Analysis

Fourier transform infrared spectroscopy (FTIR) was used to evaluate the molecular interactions between cysteine and the nanoparticle surface.

The Fe–O vibration bands characteristic of magnetite were observed near 550 cm^{-1} and 450 cm^{-1}, confirming the presence of iron oxide nanoparticles.

Additional vibrational modes associated with cysteine and cystine were identified, including bands corresponding to carboxylate groups (COO^{-}), N–H stretching vibrations, and disulfide bonds (S–S) formed by cysteine dimerization.

These results confirm the successful functionalization of SPIONs with cysteine.

Figure S6. FTIR spectra of pure SPIONs (Fe_3O_4), L-cysteine, and SPION-Cys nanoparticles.

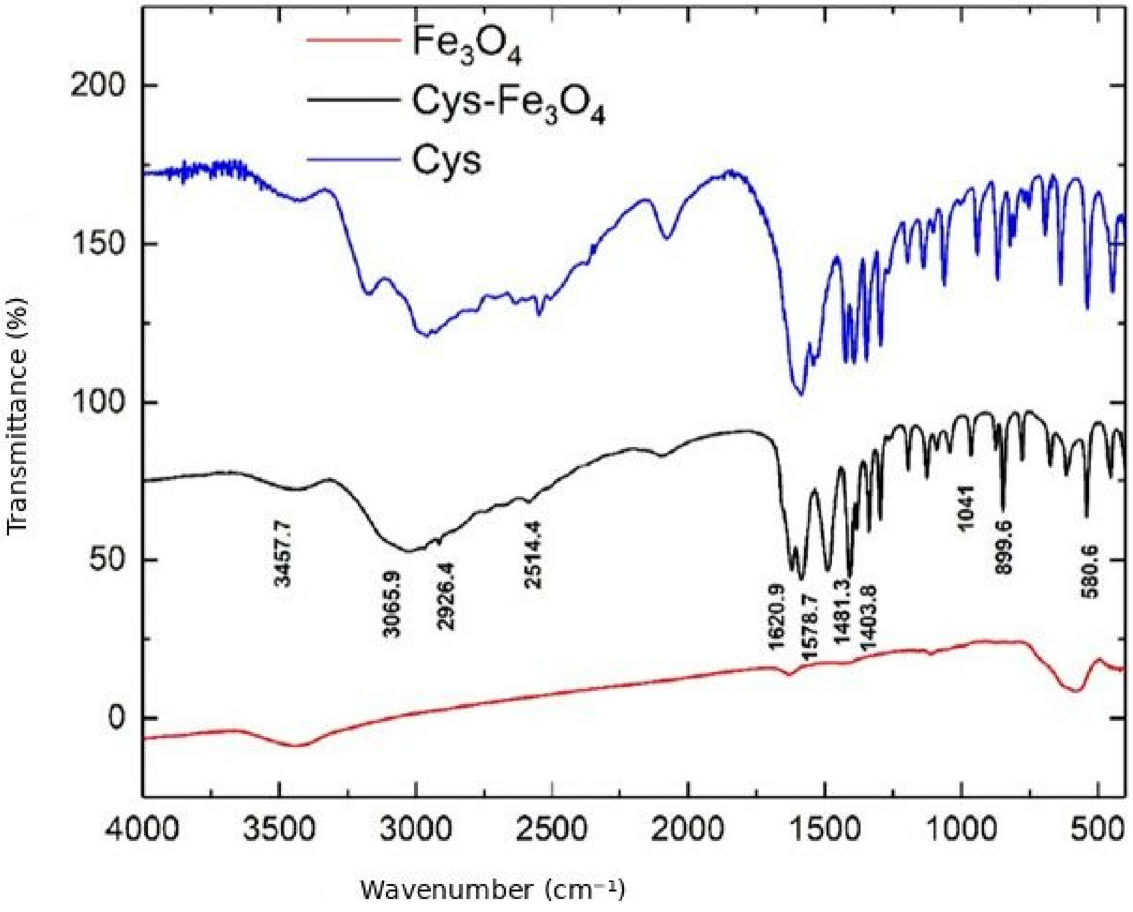

Figure S7. FTIR spectra of SPION-Cys nanoparticles synthesized for 1 h with different SPION:Cys mass ratios.

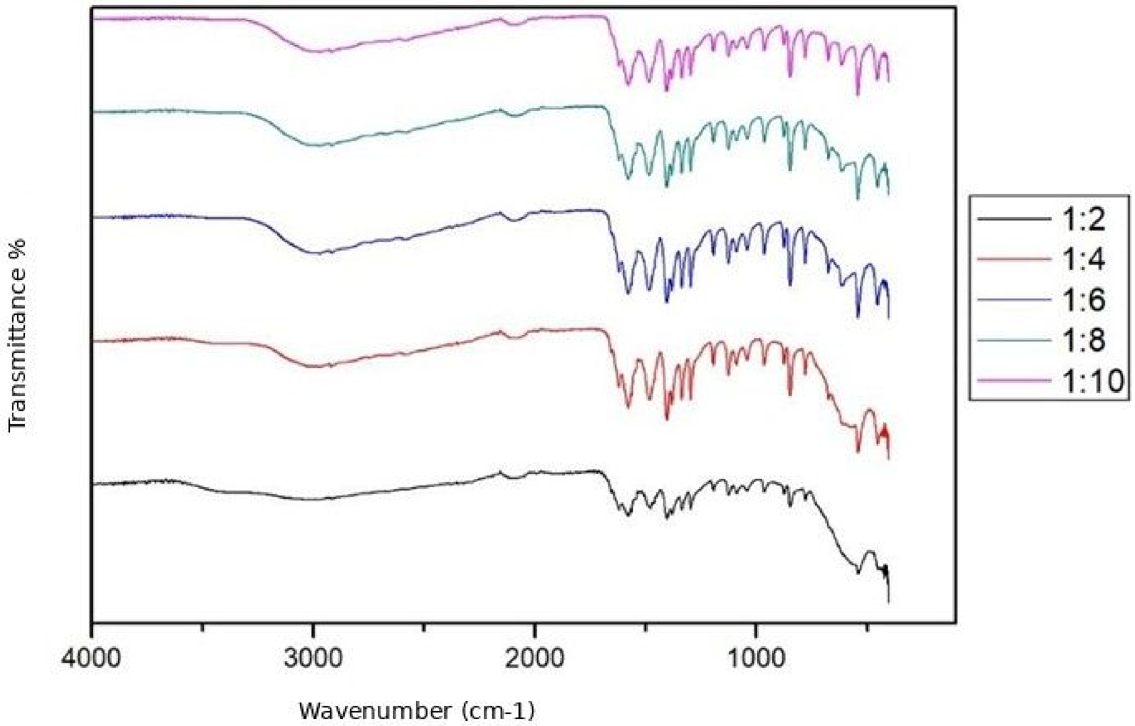

Figure S8. FTIR spectra of SPION-Cys nanoparticles synthesized for 3 h with different SPION:Cys mass ratios.

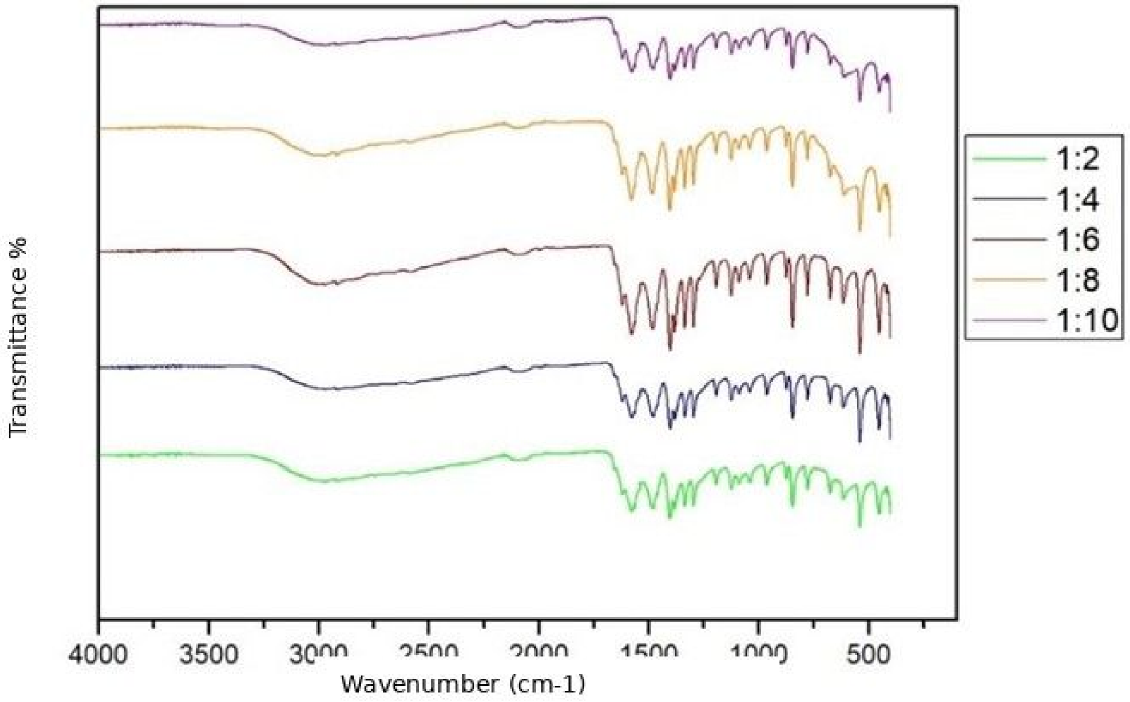

Figure S9. FTIR spectra of SPION-Cys nanoparticles synthesized for 5 h with different SPION:Cys mass ratios.

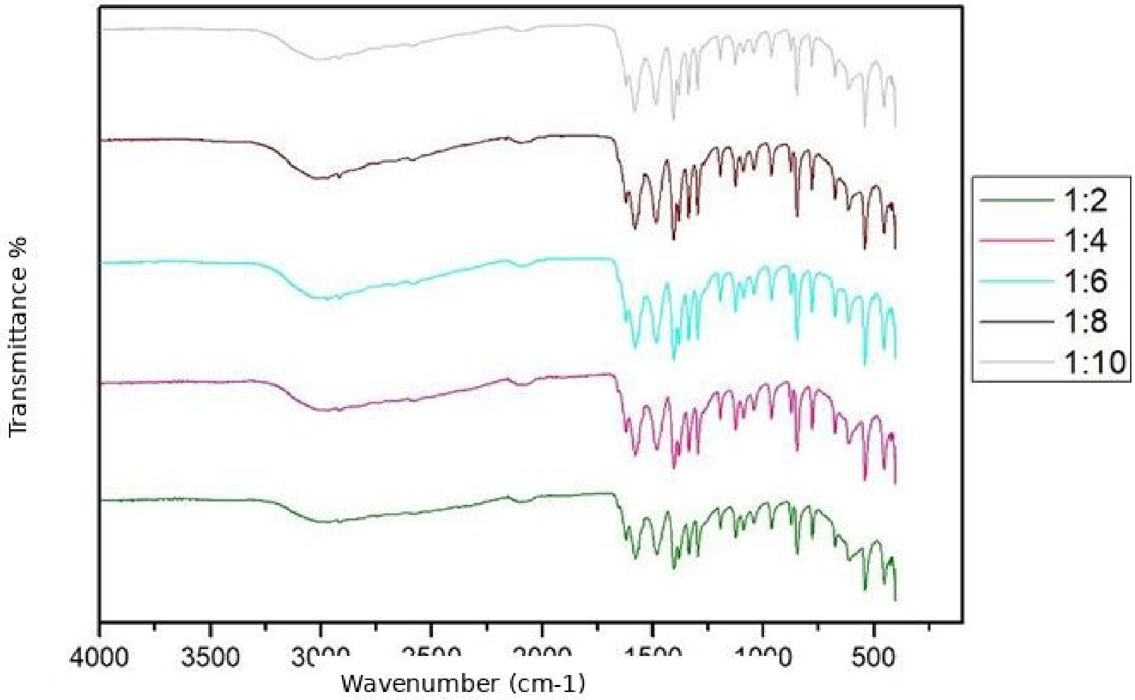

S5. Quantification of Surface Thiol Groups

The concentration of free thiol groups present on the nanoparticle surface was determined using Ellman's reagent (DTNB).

DTNB reacts with thiol groups producing the yellow anion TNB^{2-}, which exhibits a characteristic absorption band at 412 nm, allowing spectrophotometric quantification.

A calibration curve was constructed using pure cysteine solutions to determine the concentration of thiol groups present on the SPION-Cys nanoparticles.

The results demonstrated that the amount of free thiol groups varies according to the reaction time and SPION:Cys mass ratio, indicating a balance between cysteine adsorption and cystine formation.

Figure S10. Calibration curve obtained using L-cysteine for thiol quantification with Ellman's reagent (DTNB).

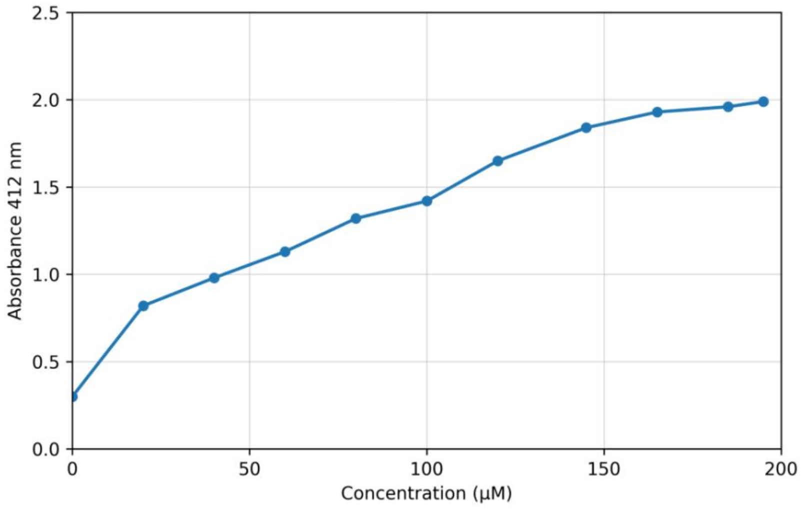

S6. Magnetic Characterization

Magnetic hysteresis curves were obtained to evaluate the magnetic behavior of the nanoparticles.

The absence of coercivity and remanent magnetization confirms the superparamagnetic behavior of the synthesized nanoparticles at room temperature.

The saturation magnetization of SPIONs was approximately 71 emu g^{-1}, and a slight reduction in magnetization was observed after cysteine functionalization due to the presence of the organic coating.

Despite this reduction, the nanoparticles maintained their superparamagnetic properties, which are essential for applications involving magnetic targeting.

S7. Hydrodynamic size, size distribution and zeta potential

The DLS measurements were performed using an ALV/CGS-3 compact goniometer system consisting of a 22 mW HeNe linearly polarized laser operating at a wavelength of λ = 633 nm, an ALV 7004 digital correlator and a pair of avalanche photodiodes operating in the pseudo-cross-correlation mode. The Cys-Fe3O4 sample was loaded in 10-mm-diameter glass cells, dispersed in deionized water using a concentration of 1 mg mL−1 and diluted in 20 mL of water (saline solution) and maintained at a constant temperature of (25 ± 1) °C. Autocorrelation functions were obtained in the angular region between 30° and 150° and adjusted by using the cumulant method

The synthesis of Cys-Fe_3O_4 produced a system with low degree of aggregation, thus resulting in high stability. The quantitative data related to the scattering characterization is displayed in Table 1.

MNps	R_H (nm)	μ_2/Γ^2	Z (mV)
**Cys-Fe_3O_4**	80.0 ± 1.2	0.26 ± 0.04	−30.0 ± 0.3

As well-established, higher hydrodynamic sizes of nanoparticles measured by DLS, in comparison with the sizes obtained by XRPD and TEM, are attributed to the presence of extra hydrate layers in aqueous environments [47]. It is worth noting that a PDI value <0.2 indicates a homogenous and monodisperse population of nanoparticles [48]. The zeta potential of Cys-Fe_3O_4 nanoparticles (−30.0 ± 0.3) mV indicates high colloidal stability [9].

Figure S11. Magnetic hysteresis curves of SPION-Cys nanoparticles functionalized with cysteine for 1 h.

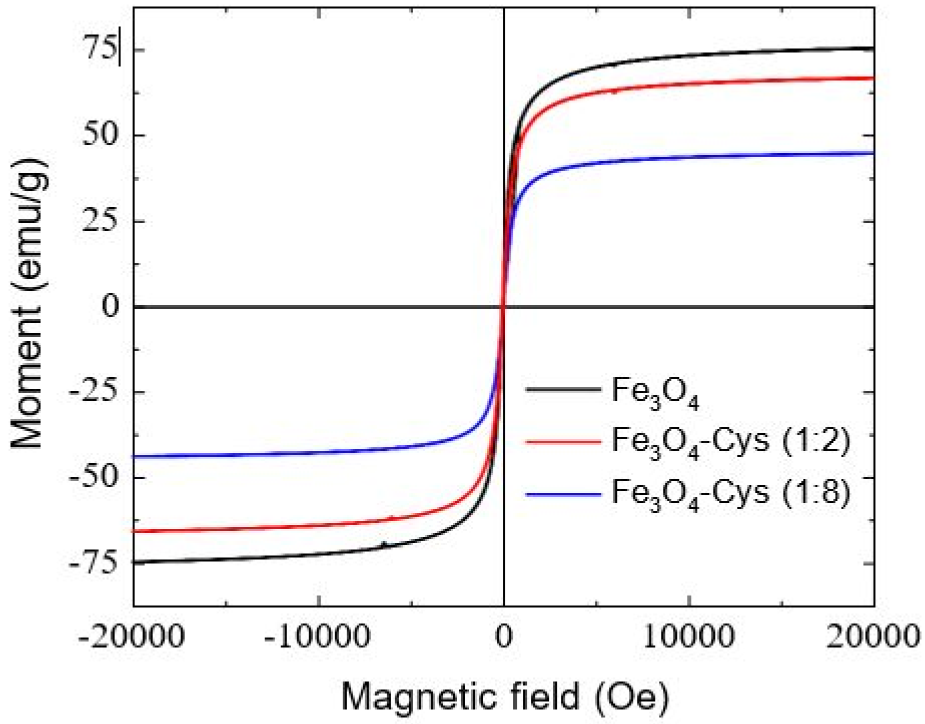

S7. In Vitro Cytotoxicity Assays

The cytotoxicity of SPIONs, cysteine, and SPION-Cys nanoparticles was evaluated in mononuclear cells and Ehrlich ascitic tumor cells.

The results demonstrated low cytotoxicity in healthy mononuclear cells at concentrations below 50 μg mL^{-1}, while tumor cells exhibited higher resistance to treatment.

These findings suggest that the SPION-Cys system presents favorable biocompatibility and supports its investigation as a magnetically responsive nanosystem with redox-associated biological activity. These in vitro data do not, by themselves, demonstrate drug delivery, tumor-specific accumulation, or a definitive antitumor mechanism

Figure S12. Cell mortality of mononuclear cells incubated for 24 h with SPIONs, cysteine, and SPION-Cys nanoparticles under normal and nutrient-deprivation conditions.

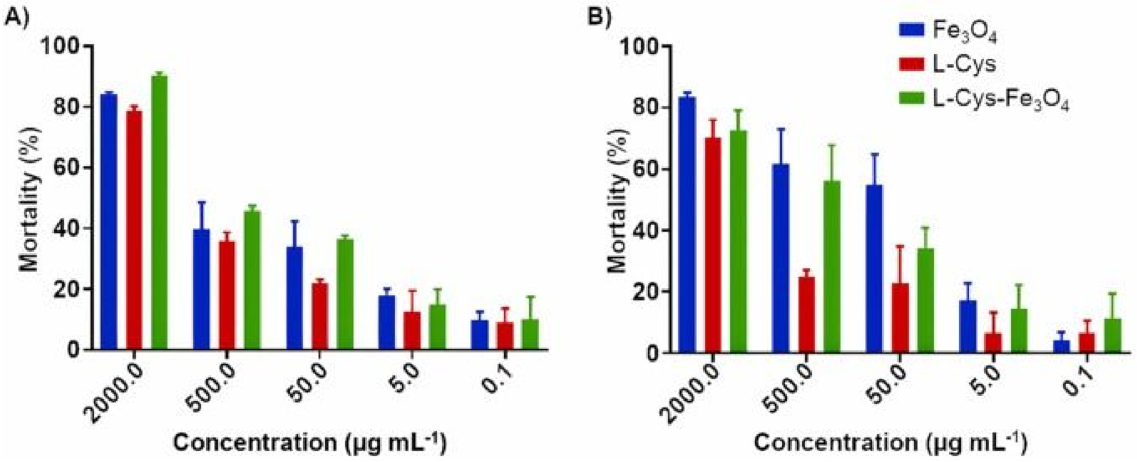

Figure S13. Cell mortality of Ehrlich ascitic tumor cells incubated for 24 h with SPIONs, cysteine, and SPION-Cys nanoparticles.

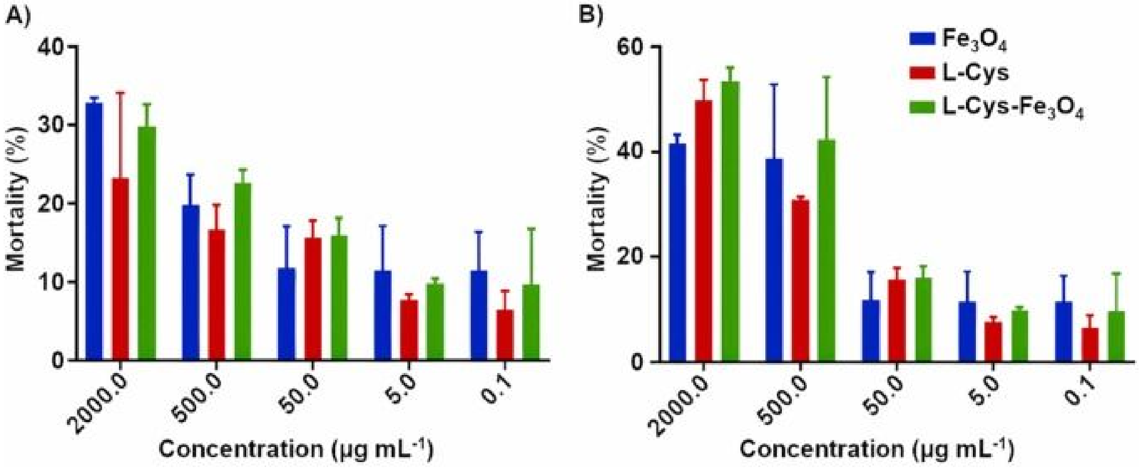

References

1. Britos, T. N.; Castro, C. E.; Bertassoli, B. M.; Petri, G.; Fonseca, F. L. A.; Ferreira, F. F.; Haddad, P. S. In Vivo Evaluation of Thiol-Functionalized Superparamagnetic Iron Oxide Nanoparticles. *Mater. Sci. Eng. C* **2019**, *99*, 171–179. https://doi.org/10.1016/j.msec.2019.01.118.

2. Mu, L.; Gao, Y.; Hu, X. L-Cysteine: A Biocompatible, Breathable and Beneficial Coating for Graphene Oxide. *Biomaterials* **2015**, *52*, 301–311. https://doi.org/10.1016/j.biomaterials.2015.02.046.

3. Britos, T.; Santana, N.; Schumacher, M. L.; Barbosa, E.; de Espindola, A.; Chagas, C.; Fonseca, F. L. A.; Ferreira, F. F.; Haddad, P. S. Antioxidant Action of L-Cysteine Anchored on the Surface of Magnetite Nanoparticles. *Next Nanotechnol.* **2024**, *6*, 100076. https://doi.org/10.1016/j.nxnano.2024.100076.

4. Schumacher, M. L.; Britos, T. N.; Fonseca, F. L. A.; Ferreira, F. F.; Feder, D.; Fratini, P.; Petri, G.; Haddad, P. S. Superparamagnetic Nanoparticles as Potential Drug Delivery Systems for the Treatment of Duchenne Muscular Dystrophy. *Nanoscale* **2025**, *17*, 3752–3767. https://doi.org/10.1039/D4NR03407D.

5. Aldini, G.; Altomare, A.; Baron, G.; Vistoli, G.; Carini, M.; Borsani, L.; Sergio, F. N-Acetylcysteine as an Antioxidant and Disulphide Breaking Agent: The Reasons Why. *Free Radic. Res.* **2018**, *52*, 751–762. https://doi.org/10.1080/10715762.2018.1468564
